# Whole-Genome Sequencing as Tool for Investigating International Tuberculosis Outbreaks: A Systematic Review

**DOI:** 10.3389/fpubh.2019.00087

**Published:** 2019-04-17

**Authors:** Marieke J. van der Werf, Csaba Ködmön

**Affiliations:** European Centre for Disease Prevention and Control, Stockholm, Sweden

**Keywords:** whole-genome sequencing, outbreak, tuberculosis, multicountry, international, cluster, Europe

## Abstract

**Background:** Whole-genome sequencing (WGS) can support the investigation of tuberculosis (TB) outbreaks. The technique has been applied to estimate the timing and directionality of transmission and to exclude cases from an investigation. This review assesses how WGS was applied in international outbreak investigations and discusses the advantages and challenges of the application of WGS.

**Methods:** Databases were searched for reports on international TB outbreak investigations. Information was extracted on: Why was WGS applied?; How was WGS applied?; Organizational issues; WGS methodology; What was learned/what were the implications of the WGS investigation?; and challenges and lessons learned.

**Results:** Three studies reporting on international outbreak investigations were identified. Retrospective WGS sequencing was performed in all studies and prospective typing in two to study TB transmission. In one study, WGS data were produced centrally (i.e., in one laboratory) and analysis was done centrally. In two studies, WGS data production was done in a decentralized manner, and analysis was centralized in one laboratory. Three groups of professionals were involved in the international outbreak investigation: public health authorities, laboratory experts, and clinicians. The reported WGS methodology applied differed between the studies in some aspects, e.g., sequencing platform; quality measures, percentage of the reference genome covered, and the mean genomic coverage; analysis, use of a reference genome or *de novo* assembly; and software used for alignment and analysis. In all three studies, in-house scripts were used for variance calling, and the single nucleotide polymorphism (SNP) approach was used for analysis. All outbreak investigation reports stated that WGS refuted suspected transmission events and provided supporting evidence for epidemiological data. Several challenges were reported of which most were not related to WGS. The only challenge related to WGS was the timeframe of getting WGS data if WGS is not routinely performed.

**Conclusions:** WGS was considered a useful addition in international TB outbreak investigations. Further standardization of the WGS methodology and good structures for international collaboration and coordination are needed to take full advantage of this new technology. Whether the use of WGS results in earlier detection of cases and thus limits transmission still needs to be determined.

## Introduction

### Rationale

Tuberculosis (TB) is an infectious disease caused by the *Mycobacterium tuberculosis* complex. It is airborne and transmitted through droplet aerosols containing the bacillus. Globally it is estimated to have caused disease in 10 million people in 2017 and is one of the top 10 causes of death worldwide (1).

Investigation of TB outbreaks in TB high burden countries is often limited to the investigation of household and close contacts, especially children under the age of 5 years (2). TB control in low TB incidence countries aims at stopping TB transmission and thus focusses on investigation of TB outbreaks next to early diagnosis and treatment of TB. With international travel infectious diseases cross borders and cause disease outbreaks affecting people living in different countries. To control international disease outbreaks identification of cases in two or more countries has resulted in international disease outbreak investigations aiming at identifying additional cases and preventing further spread (3, 4). Also for TB, international outbreak investigations have been conducted (5–8).

The International Health Regulations (IHR) oblige countries to notify the World Health Organization of all events which may constitute a public health emergency of international concern within 24 h of assessment (9). In the European Union a similar system was created in 1998, the Early Warning and Response System, which is a tool with restricted access for monitoring public health threats (10). International TB outbreak investigations have started with a notification in EWRS (7). Both the IHR notification system and the EWRS allow for early notification and bring into permanent communication competent public health authorities in countries and others responsible for determining the measures, which may be required to protect public health.

The World Health Organization defines a disease outbreak as the occurrence of disease cases in excess of normal expectancy^1^ Before the availability of molecular typing, outbreaks were defined as two or more TB cases with known exposure to each other by sharing enclosed airspace in the same period. Currently, information from molecular typing is added to epidemiological information to confirm linkage between patients.

Molecular typing methods for TB include IS*6110* restriction fragment length polymorphism, spoligotyping and mycobacterial interspersed repetitive units–variable number tandem repeat (MIRU-VNTR). These methods have been applied to outbreak investigations and provided useful additional information for TB control (5, 11–13). Since the complete genome sequence of *M. tuberculosis* was first described in 1998 (14), whole-genome sequencing (WGS) has been added to the toolbox for outbreak investigation. Several studies showed that WGS has a higher discriminatory power and subdivides clusters defined by other genotyping methods (15–17).

Recently WGS has been applied in the investigation of national outbreaks. In several investigations WGS was used to estimate the timing and directionality of transmission within clusters defined by spoligotyping and/or 24-loci MIRU-VNTR (13, 18–21); however, not always successfully (19, 20). In another investigation of an outbreak of extensively drug-resistant TB (XDR-TB) in London, the use of WGS confirmed the link between cases and guided early patient treatment (22). WGS has also helped in excluding cases from an investigation and thus to focus resources on the investigation of cases that were more likely to have been part of the transmission network (23). With WGS becoming more widely applied experience with using WGS for national outbreak investigations will quickly grow (24). WGS has also been applied for the investigation of international cross-border TB outbreaks (6–8). However, the added value of WGS for outbreak investigations remains unclear.

#### Objectives

- To assess how WGS has been applied in international TB outbreak investigations; and
- To determine the advantages and challenges of the application of WGS in international TB outbreak investigations.

#### Research Question

Is WGS a useful tool for international TB outbreak investigations, and what are the advantages and challenges?

## Methods

### Study Design

In this systematic review, we examined studies reporting on an international *M. tuberculosis* complex outbreak investigation in humans using WGS. We included all study types in all types of populations.

### Systematic Review Protocol

The review protocol was registered in PROSPERO, registration number CRD42018107259.

### Search Strategy

The search strategies combined the concepts of WGS with surveillance/outbreak and TB and was set up on 13 August 2018 (Appendix 1). Controlled vocabulary (i.e., MeSH and Emtree terms) and natural vocabulary (i.e., keywords) in multiple field search combinations were used to represent the concepts in the search strategies. Automatic email updates were set up in all the databases to continue receiving new results from the designed searches. These alerts were monitored until 5 February 2019. Additional supplementary searches have been performed by backward and forward citation chasing of the included references on 4 February 2019. No language or date restrictions were applied.

### Data Sources

We searched PubMed, EMBASE, and Scopus.

### Eligibility Criteria

Records were eligible for inclusion if they reported on a study in humans, covered *M. tuberculosis* complex, applied WGS, and the outbreak investigation was performed by two or more countries. We included all study types in all types of populations.

### Study Selection

Studies were imported into an EndNote X7 database and duplicates were removed. MW and CK independently screened the titles and abstracts to identify potentially eligible studies. The full text of potentially eligible studies was reviewed in duplicate by MW and CK against the eligibility criteria. Discrepancies were resolved by discussion between the reviewers.

### Data Extraction

MW extracted data from selected studies using a predefined data extraction form. CK checked the data extraction. Inconsistencies were resolved by discussion. For each study, we extracted the author name, year, and countries involved in the outbreak investigation. Thereafter, we extracted information on: Why was WGS applied?; How was WGS applied?; Organizational issues; WGS methodology; What was learned/what were the implications of the WGS investigation?; and challenges and lessons learned. No formal study quality assessment was performed, as any description of an international outbreak investigation was relevant for our review with the main limitation of studies being that not all areas of interest were described as is reported in the results.

### Definitions

We defined an international outbreak investigation as activities undertaken to establish the existence of an outbreak, describe the outbreak, and to identify the source, transmission mechanism, and contributory factors, as a basis for outbreak response involving two or more countries [adjusted from (25)].

### Data Analysis

We summarize the extracted information using the themes: reason for WGS; WGS application; WGS methodology; organizational issues; implications of the WGS investigation; and challenges and lessons learned.

### Ethics Statement

This review used published data and ethical review was not required.

## Results

### Study Selection

**Figure 1:**
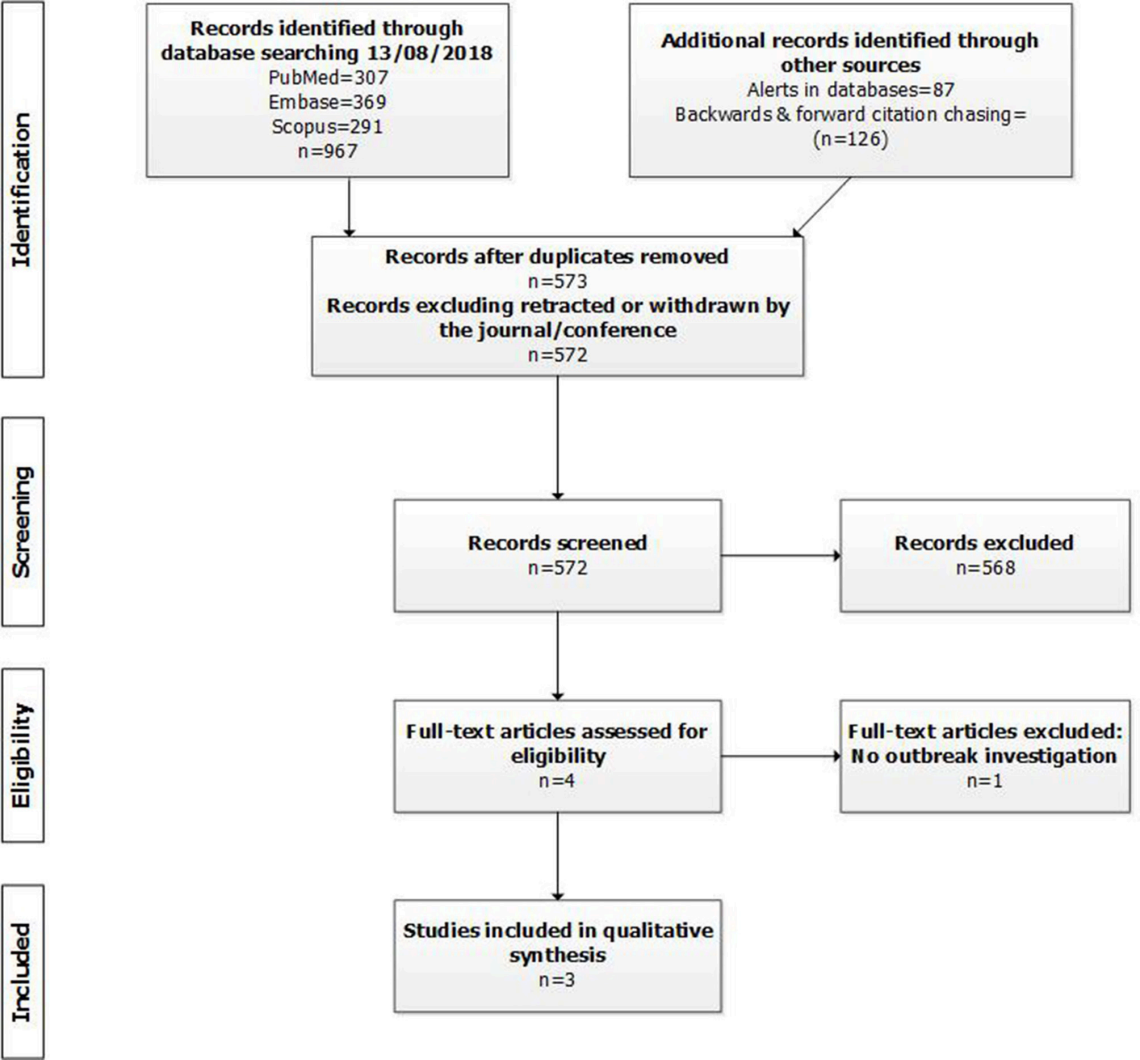
PRISMA flowchart.

The search strategy identified 572 unique records (Figure 1). Of these four were selected based on title and abstract. Studies were excluded because they did not cover: humans (39 records); *M. tuberculosis* complex (55 studies); WGS (140 studies); outbreak investigation (262); or two or more countries (69 studies). Three records were errata. After the full text assessment, three records fulfilled the eligibility criteria. One record was excluded from further analysis because it was not an outbreak investigation (17).

### Synthesized Findings

The included studies reported on outbreak investigations involving European countries and Israel and covered three (6), four (8), and 12 (7) countries. The outbreak investigations included patients diagnosed between 2010–2014, 2015–2016, and 2016–2017, respectively.

#### Reason for WGS

In all three outbreak reports WGS was applied to study TB transmission. Walker et al. (7) further specified that the aim of the outbreak investigation, including the application of WGS, was to elucidate the origin of the cluster, identify possible locations of transmission, and interrupt further transmission.

#### WGS Application

In none of the reported international outbreak investigations WGS was used as a routine investigation method for TB in all involved countries. Therefore, studies used specific criteria to select TB cases for whom WGS data needed to be collected. Criteria included a specific spoligotyping and/or 24-loci MIRU-VNTR patterns and/or drug resistance profile. Popovici et al. (8) also used place as a criterion (i.e., connected to a university in Romania). Cases identified in the contact investigation were later added to the WGS investigation. Retrospective WGS was applied in all three studies; two studies also performed prospective WGS of strains identified during the investigation (7, 8).

WGS data were produced centrally in one laboratory and analysis was done centrally in the study by Fiebig et al. (6). In the other two studies WGS data production was done in a decentralized manner and analysis was centralized in one laboratory. In addition to WGS data, all studies also collected epidemiological information including travel information.

#### Organizational Issues

In the countries included in the outbreak investigations, three groups of professionals were involved: national and local public health authorities (6–8); experts from (national reference) laboratories (6–8); and clinicians (7).

None of the studies reported on issues related to shipping of strains, e.g., method, costs, and duration. The two studies that used prospective WGS sequencing in addition to retrospective sequencing information did not report on the time till WGS results were available (7, 8). Popovici et al. (8) reported, that it takes several months before the results are available if WGS is not routinely available in the country. None of the studies in which WGS information was exchanged between countries reported on the methods or tools used for WGS data exchange.

#### WGS Methodology

In the outbreak investigation reported by Fiebig et al. (6) WGS was performed in one laboratory, whereas in the other two outbreak investigations WGS was performed in several laboratories. All but the laboratory of the Public Health Agency of Sweden used an Illumina sequencing platform (6–8), Table 1. In two outbreak investigations (6, 7) reads were mapped to the *M. tuberculosis* H37Rv reference genome, whereas in Popovici et al. (8) *de novo* assembly was used in addition to mapping against a reference genome (unspecified). Fiebig et al. (6) specified the percentage of the reference genome covered and the mean genomic coverage, i.e., at least 45 times. The percentage of the reference genome covered was not reported by the other two studies where WGS was performed in different laboratories. Walker et al. (7) aimed for a mean coverage of 20–50 times. Programs used for alignment and analysis were similar in the studies of Fiebig et al. (6) and Walker et al. (7). In all three studies, in-house scripts were used for variance calling and the analysis used a single nucleotide polymorphism (SNP) approach. The maximum difference in SNPs to define a cluster was not reported in Popovici et al. (8), and was five SNPs in Walker et al. (7) and 12 SNPs in Fiebig et al. (6).

**Table 1:** Whole-genome sequencing methodology applied to international tuberculosis outbreak investigations.

**Whole-genome sequencing methodology**	**Fiebig (6)**	**Walker (7)**	**Popovici (8)**
Sequencing platform	Illumina	Illumina and Ion Torrent (Sweden)	Illumina and Ion Torrent (Sweden)
Reference genome	Mapping against *M. tuberculosis* reference strain H37Rv	Mapping against *M. tuberculosis* reference strain H37Rv	Mapping against unspecified reference genome and *de novo* genome assembly
% of the reference genome covered	>99% of reference genome	Not reported	Not reported
Coverage depth	At least 45 times	Mean 20–50 times	Not reported
Programs used for alignment and analysis	SARUMAN exact alignment tool In-house Perl scrips for variance calling Bionumerics software (Applied Maths NV, Belgium)	Burrows-Wheeler Aligner version 0.7.12-r1039; Genome Analysis Toolkit; SAMtools Custom Perl scrips for variance calling PhylML 3.1 and Bionumerics 6.7	CLC Assembly Cell v 4.4.2 In-house script
Analysis approach	SNP mapping	SNP mapping	SNP mapping
Maximum SNP or allelic difference thresholds to define cluster	12 SNPs	5 SNPs	Not reported

*SNP, single nucleotide polymorphism*.

#### Implications of WGS

According to all three studies, WGS provided useful information for the outbreak investigation (Table 2). The outbreak investigation studies reported that WGS refuted suspected transmission events based on epidemiological or MIRU-VNTR information and thus focussed the investigation. WGS also provided supporting evidence for epidemiological data. Walker et al. (7) reported that WGS helped in identifying the direction of transmission and in identifying additional links/missing cases. Furthermore, it provided information about the origin of the strain and where transmission is likely ongoing.

**Table 2:** Implications of whole-genome sequencing in international tuberculosis outbreak investigations.

**Implications of WGS investigation**	**Fiebig (6)**	**Walker (7)**	**Popovici (8)**
Guiding contact investigation	No^*^	No	No
Identification of possible direction of transmission	No	Yes	Not reported
Identification of additional links or missing cases	No	Yes	Not reported
Identification of places of transmission	No	Yes	Not reported
Refuting suspected transmission based on epidemiological or MIRU-VNTR information	Yes	Yes	Yes
Supporting evidence for information from epidemiological data	Yes	Yes	Yes
Successful control of the outbreak	Not reported	Not reported	Not reported
Changes in TB prevention and control practices or TB laboratory and surveillance systems	Not reported	Not reported	Not reported

*MIRU-VNTR, Mycobacterial interspersed repetitive units-variable number tandem repeat; TB, Tuberculosis; WGS, Whole-genome sequencing*.

* *Contact investigation was completed before initiation of the international outbreak investigation*.

None of the studies provided evidence that WGS was essential for successful control of the outbreak. Also, no changes in TB prevention and control practices or in TB laboratory and surveillance were reported.

#### Challenges and Lessons Learned

Several challenges were reported in the three international TB outbreak investigations of which most were not related to WGS. First, the collected information on epidemiological links was difficult to interpret and it was often not known whether absence of a link meant that there was indeed no link, or that it was unknown or not reported (6). Also, a challenge in transferring patient reports was noted (6). Collection of travel information is often not a routine component of an outbreak investigation, and it was reported as challenging (7). The only challenge specifically related to WGS was the timeframe of getting WGS data, if WGS is not routinely performed for tuberculosis strains in the country. Experience from the outbreak investigation reported by Popovici et al. (8) showed that it took several months to get the WGS results and to have a link confirmed.

The main lesson learned in all three outbreak investigations was the importance of establishing collaboration and coordination between institutions in different countries involved in the investigation. This also needs a secure system for the exchange of patient data among the involved countries.

## Discussion

### Summary of Main Findings

Three studies reporting on international TB outbreak investigation using WGS were identified. The WGS methodology used for the outbreak investigation, i.e., sequencing platform, quality indicators such as genomic coverage, and scripts for variance calling, differed to some extent. In addition, the maximum difference in SNPs to define a cluster was different in the two studies that reported SNPs thresholds. WGS was a useful tool for international TB outbreak investigations according to the three studies. However, none of the studies provided evidence that WGS was essential for successful control of the outbreak or provided evidence on the cost-effectiveness of WGS for international outbreak investigations.

#### Reason for WGS

By applying WGS in international outbreak investigations researchers and experts hoped to obtain additional information on transmission that would help in controlling the outbreak. WGS can provide more information than any of the other typing methods used for studying TB transmission since it has a higher resolution. It has been shown that WGS can divide clusters identified by other methods into sub-clusters (16, 17), and can identify transmission missed by conventional epidemiological investigations (26). Furthermore, WGS can provide supporting evidence, complementary to temporal- and contact tracing data, to identify the most likely direction of transmission (27, 28).

#### WGS Application

WGS was not a standard typing method in all countries involved in the international outbreak investigations (6–8). Thus, WGS information was not readily available for all cases and WGS had to be done specifically for strains suspected to be part of the outbreak. This required a decision on the type of cases for which WGS information was to be collected. Restricting the collection of WGS information to specified cases introduces a risk of missing transmission events. This risk might be relatively low if the cases are selected based on a specific MIRU-VNTR pattern. A population based study from the Netherlands reported 86% concordance between MIRU-VNTR and WGS, although the percentages of cases clustered by MIRU-VNTR was almost twice as high (25% by MIRU-VNTR vs. 14% by WGS). In addition, clustering was only shown by WGS and not by MIRU-VNTR for 8 of 76 isolates included in the WGS cluster (29). These potential transmission events would thus have been missed, if the cluster definition was based on MIRU-VNTR pattern only.

Recently, the number of laboratories able to perform WGS has increased rapidly in the European Union (30), providing more countries access to WGS and facilitating WGS of all identified TB cases. Therefore, application of criteria for selecting cases for WGS and thus potentially excluding cases may not be needed anymore in the near future.

#### Organizational Issues

International TB outbreak investigations require the involvement of different types of stakeholders. All three outbreak investigation studies described the involvement of public health authorities and laboratory experts. Depending on how the (public) health system in countries is organized an international outbreak investigation will need the involvement of both national and local level public health authorities.

All three studies concluded that collaboration and coordination between all institutions involved in the investigation is essential. Our organization, the European Centre for Disease Prevention and Control (ECDC) was involved in the coordination of two of the international outbreak investigations (7, 8). Given the mandate of ECDC, i.e., supporting the response to public health threats in the European Union (31), this supra national organization can play a role in the coordination of outbreak investigations next to other organizations such as the World Health Organization. To ensure effective and efficient international collaboration, mechanisms for collaboration and communication should be further developed.

To be able to rapidly and efficiently investigate potential international TB outbreaks mechanisms for exchange of samples and/or data (including patient data) should be in place. None of the included studies reported on mechanisms for sample or data exchange. Given that patient information would need to be exchanged these exchanges need to be done in a secure way ensuring that data protection and privacy regulations such as the European Union regulation 2016/679 on the protection of natural persons with regard to the processing of personal data and on the free movement of such data (32) are adhered to. Currently, data can be exchanged among European Union countries in a secured way through the Early Warning and Response System (33, 34). Communication between countries can also be done in the framework of the International Health Regulation (9).

#### WGS Methodology

In two studies (7, 8) WGS data production was done in several laboratories. Since WGS is not standardized for TB (35) this entails a risk that WGS data produced by different laboratories are not 100% comparable. All laboratories will start with genomic DNA from *M. tuberculosis* but may use different protocols for library construction prior to sequencing and library preparation methodology has been shown to play an important role in WGS data quality and may thus influence the results (36, 37). Also, data analysis and interpretation has not been standardized.

Some WGS quality control indicators have been proposed and used (38). These include assessment of the quality of genomic DNA, average depth of genome coverage, and percent of reference genome covered. Fiebig et al. (6) reported on quality targets for percent of the reference genome covered and coverage depth, whereas the two studies that had WGS performed in different laboratories did not report on specific quality targets (7, 8). To ensure comparability of data generated by different laboratories to enable the investigation of outbreaks that go beyond the coverage area of one laboratory there is a need for minimal set of quality standards. The EU wide project EUSeqMyTB (35) will develop the minimal set of standards for WGS methodology to be used in routine European Union level TB molecular surveillance activities.

To ensure that results from laboratory tests are of high quality, reliable, and comparable, external quality assessment is used. Within the European Union, the European Reference Laboratory Network for TB organizes external quality assessment for TB diagnosis and resistance testing and for MIRU-VNTR (39, 40). Recently, the Network established an external quality assessment scheme for WGS. A first pilot was performed in 2015 using five samples with known mutations in genes associated with drug resistance. Participating laboratories were asked to report all the mutations detected in these genes and the results were compared to the results of the reference laboratory. In this first pilot study, most laboratories missed a number of mutations that had been identified by the reference laboratory and found a variety of additional mutations not found by the reference laboratory. In the second WGS external quality assessment round in 2016 participating laboratories were asked to report the WGS data they felt important. The results showed that reporting of mutations at specified loci identified as significantly associated with drug resistance was highly diverse by the participating laboratories. In 2017, participants were asked to identify any mutations strongly associated with drug resistance and to report their position in the respective gene. In addition, laboratories were asked to identify DNA specimens they considered either identical or genetically closely related. This round was also the first where the WGS external quality assessment results were scored and certificates issued. The external quality assessment scheme for TB WGS developed by the European Reference Laboratory Network for TB seems to be one of the first attempts for assessing the quality of WGS for a specific pathogen although the need for external quality assessment or proficiency testing for WGS of pathogens has been identified earlier (41, 42). In general, only few experiences with external quality assessment schemes for WGS have been published (43, 44).

In the framework of the EUSeqMyTB pilot project (35) a comparison of different WGS analysis pipelines was undertaken using fastq files from a well-defined set of isolates. This analysis showed that some pipelines identify more SNPs then others. The main question that needs to be answered is whether different analysis pipelines result in different conclusions about transmission and relatedness.

In the identified international outbreak investigations, analysis of WGS data was performed centrally in one of the participating laboratories using a SNP-based in-house analysis pipeline. The use of in-house analysis pipelines prohibits easy comparison of results between studies. An alternative approach would be the use of a common nomenclature based on a standardized allele numbering system, which would facilitate exchange of information. For TB a core genome multilocus sequence typing (cgMLST) has been proposed and a web-based nomenclature server is available (45).

#### Implications of WGS

All three studies reported on advantages of using WGS in international outbreak investigations. Applying WGS in national outbreak investigations has also shown benefits. In the UK, it was shown that using WGS in a multidrug-resistant TB outbreak investigation allowed to exclude one-third of cases from the investigation (23). Resources could thus be focussed. Use of WGS has allowed verification of clusters, i.e., it confirmed that cases were part of a single transmission chain (19) and it identified missed transmission events (26). Reports on national outbreak investigations have also shown that WGS can indicate the direction of transmission (21, 28, 46). However, this does not seem to be the case in all settings (20).

#### Challenges and Lessons Learned

The main challenges reported in the three international outbreak investigations (6–8) were related to the collection and exchange of epidemiological and travel information. Above we discuss solutions for these challenges. If WGS was not routinely performed, it did take considerable time to get the WGS results since re-culturing of samples was required (8). The increase in the number of countries that have access to WGS may result in routine performance of WGS on all TB samples and thus timely availability of the information (30).

## Limitations

We aimed to collect and abstract information from studies reporting on international outbreak investigations using WGS on: Why was WGS applied?; How was WGS applied?; Organizational issues; WGS methodology; What was learned/what were the implications of the WGS investigation?; and challenges and lessons learned. We searched PubMed, EMBASE, and Scopus but did not search for studies reporting on international outbreak investigations using WGS in the gray literature. We therefore might have missed studies reporting on the application of WGS in international TB outbreak investigations and thus not have identified all information on Why was WGS applied?; How was WGS applied?; Organizational issues; WGS methodology; What was learned/what were the implications of the WGS investigation?; and challenges and lessons learned.

We used a detailed data collection tool. Not all information was reported by the included studies (Tables 1, 2). This may bias our analysis. Furthermore, only three studies were identified that reported on the use of WGS for international outbreak investigations. Since outbreak investigations are a routine activity for public health experts, more international outbreak investigations may have used WGS without being published in (scientific) reports. Thus, some important experiences may have been missed in this analysis.

## Conclusions

WGS seems to be a promising tool for international outbreak investigations. WGS methodology needs to be standardized further, especially quality control, analysis, and interpretation, to better support cross-border collaboration in outbreak investigations. It also allows for the prediction of drug resistance and therefore testing practices have already changed in some countries (24), making WGS information also available for outbreak investigations. However, the advantages for public health still need to be determined. More specifically, does the use of WGS result in earlier detection of cases, which belong to the same transmission chain, and thus limit transmission and result in smaller clusters?

## Author Contributions

MW designed the systematic review, performed the systematic review, analyzed the results, and wrote the manuscript. CK performed the systematic review and critically reviewed the manuscript.

### Conflict of Interest Statement

The authors declare that the research was conducted in the absence of any commercial or financial relationships that could be construed as a potential conflict of interest.

## Footnotes

^1^http://www.who.int/environmental_health_emergencies/disease_outbreaks/en/.
