# Supplementary material for: Whole-Genome Sequencing as Tool for Investigating International Tuberculosis Outbreaks: A Systematic Review
Source: Front Public Health. 2019 Apr 17;7:87. doi: 10.3389/fpubh.2019.00087 (PMC6478655; doi:10.3389/fpubh.2019.00087)
Supplement: Supplementary file 1 [file Table_1.pdf]

Appendix 1: Search strategy

PubMed search strategies (run 13/08/2018)

Search	Query	Results
**#1**	Search ("Whole Genome Sequencing"[Mesh] OR wgs[TW] OR whole genome sequenc*[TW] OR whole genomic sequenc*[TW] OR whole genome multilocus sequenc*[TW] OR whole genome referenc*[TW] OR full genome sequenc*[TW] OR full genomic sequenc*[TW] OR entire genome sequenc*[TW] OR entire genomic sequenc*[TW] OR complete genome sequenc*[TW] OR complete genomic sequenc*[TW] OR ((whole[TI] OR entire[TI] OR full[TI] OR complete[TI]) AND genom*[TI] AND (sequenc*[TI] OR referenc*[TI])) OR ((Whole[OT] OR entire[OT] OR full[OT] OR complete[OT]) AND genom*[OT] AND (sequenc*[OT] OR referenc*[OT])) OR next-generation sequenc*[TW] OR ngs[TW] OR (next[TI] AND generation[TI] AND sequenc*[TI]) OR (next[OT] AND generation[OT] AND sequenc*[OT]) OR "High-Throughput Nucleotide Sequencing"[Mesh] OR High-Throughput Nucleotide Sequenc*[TW] OR High-Throughput Sequenc*[TW] OR (high[TI] AND Throughput[TI] AND sequenc*[TI]) OR (high[OT] AND Throughput[OT] AND sequenc*[OT]) OR massive parallel sequenc*[TW] OR (massiv*[TI] AND parallel*[TI] AND sequenc*[TI]) OR (massiv*[OT] AND parallel*[OT] AND sequenc*[OT]) OR deep sequenc*[TW] OR (deep*[TI] AND sequenc*[TI]) OR (deep*[OT] AND sequenc*[OT]))	70892
**#2**	Search ("Disease Transmission, Infectious"[Mesh] OR "Disease Outbreaks"[Mesh] OR "Epidemics"[Mesh] OR "Population Surveillance"[Mesh] OR "Epidemiological Monitoring"[Mesh] OR transmi*[TW] OR outbreak*[TW] OR pandemic*[TW] OR spread*[TW] OR epidemic*[TW] OR endemic[TW] OR surveillance*[TW] OR cluster detect*[TW] OR clusters detect*[TW] OR cluster identificat*[TW] OR clusters identificat*[TW] OR ((detect*[TI] OR identificat*[TI]) AND cluster*[TI]) OR ((detect*[OT] OR identificat*[OT]) AND cluster*[OT]) OR linked case*[TW] OR linked isolate*[TW] OR (link*[TI] AND (case*[TI] OR isolat*[TI])) OR (link*[OT] AND (case*[OT] OR isolat*[OT])))	1152580
**#3**	Search (genomic epidemiolog*[TW] OR genomic surveillance*[TW] OR genome surveillance*[TW] OR genome epidemiolog*[TW] OR (genom*[TI] AND (epidemiolog*[TI] OR surveillance*[TI])) OR (genom*[OT] AND (epidemiolog*[OT] OR surveillance*[OT])))	1541
**#4**	Search ("Tuberculosis"[Mesh] OR tuberculos*[TW] OR tb[TW] OR mtbc[TW])	254276
**#5**	Search (#1 AND #2 AND #4)	272
**#6**	Search (#3 AND #4)	62
**#7**	Search (#5 OR #6)	307

Embase.com search strategies (run 13/08/2018)

Search	Query	Results
**#1**	(((whole OR entire OR full OR complete) NEAR/3 genom* NEAR/3 (sequenc* OR referenc*)):ab,ti) OR ((next NEXT/5 generation* NEXT/5 sequenc*):ab,ti) OR ngs:ab,ti OR wgs:ab,ti OR 'high throughput sequencing'/exp OR ((high NEAR/3 throughput NEAR/3 sequenc*):ab,ti) OR ((massiv* NEAR/3 parallel NEAR/3 sequenc*):ab,ti) OR ((deep NEAR/3 sequenc*):ab,ti)	97782

Search	Query	Results
**#2**	'surveillance'/exp OR 'disease surveillance'/exp OR 'disease transmission'/exp OR 'epidemic'/exp OR surveillance*:ab,ti OR transmi*:ab,ti OR outbreak*:ab,ti OR pandemic*:ab,ti OR spread*:ab,ti OR epidemic*:ab,ti OR endemic:ab,ti OR ((link* NEAR/3 (case* OR isolat*)):ab,ti) OR ((cluster* NEAR/5 (detect* OR identificat*)):ab,ti)	1236683
**#3**	(genom* NEAR/5 (epidemiolog* OR surveillance*)):ab,ti	2238
**#4**	'tuberculosis'/exp OR tuberculos*:ab,ti OR tb:ab,ti OR mtbc:ab,ti	306730
**#5**	#1 AND #2 AND #4	332
**#6**	#3 AND #4	64
**#7**	#5 OR #6	369

Scopus search strategies (run 13/08/2018)

Search	Query	Results
**#1**	TITLE-ABS (((whole OR entire OR full OR complete) W/3 genom* W/3 (sequenc* OR referenc*)) OR (next AND next/5 AND generation* AND next/5 AND sequenc*) OR (high W/3 throughput W/3 sequenc*) OR (massiv* W/3 parallel W/3 sequenc*) OR (deep W/3 sequenc*) OR ngs OR wgs)	69,481
**#2**	TITLE-ABS (surveillance* OR transmi* OR outbreak* OR pandemic* OR spread* OR epidemic* OR endemic OR (link* W/3 (case* OR isolat*)) OR (cluster* W/5 (detect* OR identificat*)))	2,606,496
**#3**	TITLE-ABS (genom* W/5 (epidemiolog* OR surveillance*))	2,305
**#4**	TITLE-ABS (tuberculos* OR tb OR mtbc)	257,265
**#5**	#1 AND #2 AND #4	243
**#6**	#3 AND #4	77
**#7**	#5 OR #6	291
